# Blocking the transmission of heartworm (*Dirofilaria immitis*) to mosquitoes (*Aedes aegypti*) by weekly exposure for one month to microfilaremic dogs treated once topically with dinotefuran-permethrin-pyriproxyfen

**DOI:** 10.1186/s13071-017-2439-3

**Published:** 2017-11-09

**Authors:** John W. McCall, Elizabeth Hodgkins, Marie Varloud, Abdelmoneim Mansour, Utami DiCosty

**Affiliations:** 1 TRS Labs, 215 Paradise Boulevard, 30607 Athens, GA USA; 2 Ceva Animal Health, Lenexa, KS USA; 3 Ceva Santé Animale, 10 Avenue de la ballastière, 33500 Libourne, France

**Keywords:** Reservoir, *Dirofilaria immitis*, Transmission, Repellency, Vector, Mosquitoes

## Abstract

**Background:**

This study assessed the influence of a topical ectoparasiticide (dinotefuran-permethrin-pyriproxyfen, DPP, Vectra®3D, Ceva Animal Health) on the acquisition of heartworm microfilariae by mosquitoes exposed to microfilaremic dogs weekly for 1 month.

**Methods:**

Six beagle dogs (9.2 ± 1.6 kg body weight) infected with *Dirofilaria immitis* were allocated to two groups of three dogs: an untreated control group and a DPP-treated group. Dogs were treated on Day 0 and exposed under sedation for 1 h to 80 ± 20 unfed *Aedes aegypti*. Each dog was exposed to mosquitoes released into mosquito-proof containers on Days −7 (pretreatment), 7, 14, 21 and 28. Up to 20 engorged mosquitoes were aspirated from the cage as soon as they were blood-fed. They were dissected and the blood from each midgut was stained for a microfilaria (MF) count. After each exposure, mosquitoes were classified as live, moribund or dead and engorged or nonengorged. The number of dead mosquitoes was recorded daily for 16 days, when the live mosquitoes were dissected to count the infective third-stage larvae (L3).

**Results:**

Prior to treatment, 95% of the engorged mosquitoes in both groups had MF. After treatment, engorgement rates for the treated group were 0%, 2.3%, 2.7% and 2.2% for Days 7, 14, 21 and 28, respectively, with anti-feeding efficacy (repellency) of 100%, 98.0%, 95.8% and 97.0%, respectively. A total of 22 mosquitoes fed on treated dogs; most of them were dead within 24 h, and all were dead within 72 h. Only 2 unfed mosquitoes exposed to treated dogs survived the incubation period and no L3 were found in them. A total of 121 of the 132 (91.6%) surviving mosquitoes that had engorged on untreated dogs had an average of 12.3 L3 per mosquito (range, 0-39).

**Conclusions:**

DPP was more than 95% effective in inhibiting blood-feeding and killing both engorged and nonengorged mosquitoes exposed weekly to microfilaremic dogs for 28 days after treatment. Treatment with DPP was completely effective in killing the few mosquitoes that fed on the treated dogs before they lived long enough for the microfilariae to develop to L3 and, consequently, was completely effective in blocking the transmission of L3 to other animals. DPP can break the life cycle of *D. immitis* and prevent infected dogs and infected mosquitoes from being effective reservoirs and can slow down the spread of heartworms, even those resistant to macrocyclic lactone preventives.

## Background

Heartworm (*Dirofilaria immitis*) is a widespread filarial vector-borne disease for which dogs are the natural, definitive hosts. Cats and humans are included in a list of numerous abnormal hosts. In canine and feline hosts, the migrating parasite within the circulatory system can induce dramatic conditions that may lead to death. The vectors of this nematode are female mosquitoes that become infected by feeding on a microfilaremic animal. After development of the worm into infective third-stage larvae (L3), infected mosquitoes transmit the parasite to the next host by L3 entering the puncture wound made by the mosquito mouthparts immediately after the blood meal. Numerous species of mosquitoes, including *Aedes aegypti* [1–3], have been identified as competent vectors of *D. immitis*. While the strategy against this disease has been primarily based on chemoprophylaxis targeting the migrating stages in the dog by the use of macrocyclic lactones, less attention has been given to the arthropod vectors themselves. However, several studies have demonstrated the potential of repellents, including pyrethroids such as permethrin, in reducing mosquito bites in humans [4] and dogs [5]. Permethrin also has been used for many years for the control of ectoparasites on companion animals and farm animals.

Dinotefuran is a rapid-acting insecticidal agent with proven efficacy against insecticide-resistant mosquitoes [6]. Although in one study dinotefuran was less toxic than other more commonly used insecticides (eg, deltamethrin, carbosulfate, temephos) against the strains of *Anopheles gambiae*, *Culex quinquefasciatus*, and *A. aegypti* used in the study, the toxicity was not strongly affected by the presence of common resistance mechanisms (ie, kdr mutations and insensitive acetylcholinesterase), and the carbamate-resistant strain of *C. quinquefasciatus* was significantly more affected than the susceptible strain. Thus, the absence of cross-resistance makes neonicotinoids, such as dinotefuran, good potential candidates for vector-borne disease control, particularly in areas where mosquitoes are resistant to insecticides [6]. Pyriproxifen targets the insect endocrine system by mimicking the activity of juvenile hormone. For example, it breaks the flea life cycle by preventing development of immature stages of fleas, thereby arresting the development of flea eggs, flea larvae and pupae. A commercially available, topically applied product containing dinotefuran, permethrin and pyriproxyfen, with label indications for fleas, ticks, biting flies, sand flies and mosquitoes exhibits strong and monthlong anti-feeding and insecticidal properties against biting arthropods [7, 8]. By targeting the mosquito vectors, this combination could also prevent the uptake of microfilariae and, thus, their subsequent development to the infective stage.

This study was designed to explore the blocking effect of a dinotefuran-permethrin-pyriproxyfen topical combination on *D. immitis* microfilaremic dogs against the acquisition of microfilariae by the bite of uninfected mosquitoes.

## Methods

The study was exploratory, blinded, controlled and unicenter, and the protocol was approved by an ethics committee (IACUC) prior to its start. The products were administered to test animals by individuals who were not involved in performing the posttreatment assessments and observations. Study groups were coded to blind the assessors. The schedule of the study is described in Table 1.

**Table 1:** Study design

Study Step	Study Day				
Treatment administration	0				
Microfilaremia	−7	m^a^	14	21	28
Mosquito infestation	−7	7	14	21	28
MF^b^ count in mosquito midguts	−7	7	14	21	28
Incubation of mosquitoes	−6-9	8-23	15-30	22-37	29-44
L3 count in mosquitoes	9	23	30	37	44

^a^ *m* missing. ^b^ *MF* microfilariae

### Dogs

Six adult beagle dogs (from 6.6-11.0 kg BW) infected with *D. immitis* multi-resistant JYD-34 isolate and microfilaremic were involved in the study. To be included, the dogs had to be microfilaremic on Day −11 and not have been treated with any ectoparasiticide for at least 3 months before the start of the study. The dogs were individually identified by a tattoo and were fed commercial dog food once daily with water available ad libitum. The dogs were housed individually in an indoor kennel. During the 9-day acclimation period, the dogs were bathed with a noninsecticidal shampoo and exposed to mosquitoes on Day −7 prior to treatment. Counts were conducted to establish the feeding and survival rate of mosquitoes. The dogs were assigned to two groups of three dogs with balanced Day −7 microfilaremia levels. This was done by ranking the dogs by microfilarial count, blocking the dogs in sets of two dogs with similar counts and then randomly allocating the dogs in each set to a treated or an untreated control group (Table 2).

**Table 2:** Dogs and treatment

Group	Dog	Microfilaremia (MF^b^/20 μL blood, Day −7)	Body Weight (kg, Day −7)	DPP^a^ Volume (mL, Day 0)
Control	1	51	9.5	0
	2	39	9.3	0
	3	185	11.0	0
DPP	1	545	6.6	1.6
	2	36	8.6	1.6
	3	52	10.5	3.6

^a^DPP: dinotefuran + permethrin + pyriproxyfen (Vectra® 3D). ^b^MF: microfilariae

### Treatment

The dogs in the DPP group were treated on Day 0 with DPP (Vectra® 3D, Ceva Animal Health) containing dinotefuran (4.95% *w*/w), pyriproxyfen (0.44% w/w) and permethrin (36.08% w/w). The product was applied topically according to the label, as a line-on from the base of the tail to the shoulders. Dogs in the control group were untreated.

### Mosquito challenges

Each dog was exposed individually to 80 ± 20 unfed mosquitoes on Days −7, 7, 14, 21 and 28 of the study. The mosquitoes were 4- to 5-day-old female *A. aegypti* (Liverpool black-eyed strain) fed on water with sugar until exposure time. Dogs were sedated by IM injection of dexmedetomidine at 0.02 mg/kg BW (Dexdomitor®, Orion, Espoo, Finland) and butorphanol at 0.2 mg/kg BW (Torbugesic®, Zoetis). Each dog was placed in a dedicated mosquito-proof chamber (73.7 cm long, 40.6 cm wide and 33 cm high) into which the mosquitoes were released. Dogs were exposed to mosquitoes for 60 min. The procedure was conducted during the day and under artificial light.

Mosquito counts were conducted during aspiration at the end of the infestation by systematically examining all areas of the animal and of the cage. The mosquitoes were assessed visually and counted as live, moribund or dead and as fed or unfed. A mosquito was considered as live when it exhibited normal behavior and was capable of flying. Moribund mosquitoes were unable to perform normal locomotion and exhibited clear signs of neurological disruption. Engorgement of mosquitoes was assessed by visual inspection of individual mosquitoes, looking at redness and enlargement of the abdomen. The dead mosquitoes were preserved in vials containing ethanol (70%) and stored at −20 °C within 3 h of collection. The live and moribund mosquitoes were collected for viability assessment.

### Viability of mosquitoes and larval load

The live and moribund mosquitoes collected were incubated in a temperature (28 °C) and humidity (80%) controlled insectary for 16 days. Viability was assessed daily. After incubation in the insectary for 16 days, live mosquitoes were dissected individually for an L3 count. The dead mosquitoes collected during the incubation period and all mosquitoes not dissected at the end of the incubation period were preserved in vials with ethanol (70%) and stored at −20 °C.

### Microfilaremia of dogs

Blood was taken from each dog for a microfilarial count on Day −11 and thereafter immediately before each exposure of the dog to mosquitoes on Days −7, 14, 21 and 28. Microfilaremia was determined using a modified Knott’s test [9].

### Microfilarial load of mosquitoes

As soon as individual mosquitoes had fed, a total of up to 20 mosquitoes was collected by aspiration from each exposure chamber. The collected mosquitoes were exposed for 2 min in a freezer (−20 °C) for immobilization, and the wings and legs were removed. Only the mosquitoes for which a blood meal was completed were collected. These mosquitoes were dissected individually: the abdomen was separated and midgut contents were smeared on a slide. The slide was Giemsa-stained before microfilarial counts.

### Analysis

#### Anti-feeding effect or repellency

For each time point after exposure, the anti-feeding effect was calculated:

$$ \mathrm{Anti}\hbox{-} \mathrm{feeding} \mathrm{effect}\ \left(\%\right)=100\times \frac{\left(\mathrm{Cf}-\mathrm{Tf}\right)}{\mathrm{Cf}} $$

Where Cf was the arithmetic mean of fed female mosquitoes (live fed + moribund fed + dead fed) in the control group, and Tf was the arithmetic mean of the fed female mosquitoes in the treated group.

#### Knock-down effect

For each time point after exposure, the knock-down effect was calculated:

$$ \mathrm{Knock}\hbox{-} \mathrm{down}\  \mathrm{effect}\;\left(\%\right)=100\times \frac{\left(\mathrm{Clm}-\mathrm{Tlm}\right)}{\mathrm{Cl}} $$

Where Clm was the arithmetic mean of live (live engorged + live unengorged) in the control group, and Tlm was the arithmetic mean of the live female mosquitoes in the treated group. The knock-down effect was calculated at the end of the exposure (1 h). The knock-down effect compared only the number of live (not including the moribund) females in the treated and control groups.

#### Insecticidal effect

For each time point after exposure, the mortality effect was calculated:

$$ \mathrm{Mortality}\left(\%\right)=100\times \frac{\left(\mathrm{Cl}\hbox{-} \mathrm{Tl}\right)}{\mathrm{Cl}} $$

Where Cl was the arithmetic mean of live and moribund female mosquitoes (live engorged + live unengorged + moribund engorged + moribund unengorged) in the control group, and Tl was the arithmetic mean of the live and moribund female mosquitoes in the treated group. The mortality effect was calculated at the end of the exposure (1 h) and daily for the 16 days of the postexposure incubation.

#### Infectivity of mosquitoes

After each exposure, the infectivity of mosquitoes was calculated:

$$ \mathrm{Infectivity}\  \mathrm{effect}\ \left(\%\right)=100\times \frac{\left(\mathrm{Ci}\hbox{-} \mathrm{Ti}\right)}{\mathrm{Ci}} $$

Where Ci was the arithmetic mean of L3 collected from mosquitoes exposed to each dog in the control group, and Ti was the arithmetic mean of L3 collected from mosquitoes exposed to each dog in the treated group.

The infectivity was calculated for each day of exposure considering mosquitoes (fed and unfed) that survived the 16-day incubation period.

A total of only 22 mosquitoes fed on dogs in the treated group, compared with 810 in the untreated control group. Most of the 22 mosquitoes were dead by the second day, and all were dead by the third day. Considering the small sample size, no statistical analysis was conducted, and the individual data were reported.

### Guidelines

This study was carried out in compliance with Good Clinical Practice requirements [10]. Except for the number of dogs, the study was conducted in compliance with US EPA Product Performance Test Guidelines OPPTS 810.3300: Treatments to Control Pests of Humans and Pets.

## Results

At the beginning of the study, the dogs exhibited microfilaremia ranging from 36 to 545 microfilariae per 20 μL of blood. Microfilaremia was not assessed on Day 7. Microfilaremia was maintained in all dogs for the duration of the study (Table 3). No adverse effects to any of the treatment applications were observed in any dogs during the study.

**Table 3:** Kinetics of microfilaremia (MF/20 μL of blood) in two groups (control untreated or DPP^a^ treated on Day 0) of *Dirofilaria immitis-*infected donor dogs over 40 days

Microfilaremia Group	Dog	−11	Study Day −7	14	21	28
Control	1	38	51	173	116	189
	2	18	39	127	191	170
	3	121	185	142	193	218
DPP	1	642	545	1174	940	750
	2	8	36	116	185	184
	3	14	52	25	20	33

^a^DPP: dinotefuran + permethrin + pyriproxyfen (Vectra® 3D)

### Anti-feeding efficacy

Before treatment, mosquito engorgement rates for all dogs ranged from 78.8% to 96.7% (data not shown), and the geometric mean number of fed mosquitoes in the control group ranged from 57.6 on Day 21 to 74.6 on Day 14 (Table 4). After treatment, the geometric mean number of mosquitoes that fed on treated dogs ranged from 0 on Day 7 to 2.4 on Day 21. The anti-feeding efficacy (repellency) of DPP ranged from 95.8% on Day 21 to 100% on Day 7.

**Table 4:** Geometric mean number of blood-fed and live mosquitoes and immediate anti-feeding and knock-down efficacy of DPP^a^ (administered on Day 0) after 1 h of exposure (%) at weekly intervals over 1 month

	Control		DPP^a^		Efficacy (%)	
Day	Fed	Live	Fed	Live	Anti-feeding	Knock-down
−7	68.7	81.8	59.1	66.8	–	–
7	68.5	75.9	0	0	100	100
14	74.6	80.3	1.5	1.4	98.0	98.3
21	57.6	73.7	2.4	3.3	95.8	95.5
28	67.5	71.9	2	1.5	97.0	98.0

^a^DPP: dinotefuran + permethrin + pyriproxyfen (Vectra® 3D)

### Microfilaria uptake by mosquitoes

**Fig. 1:**
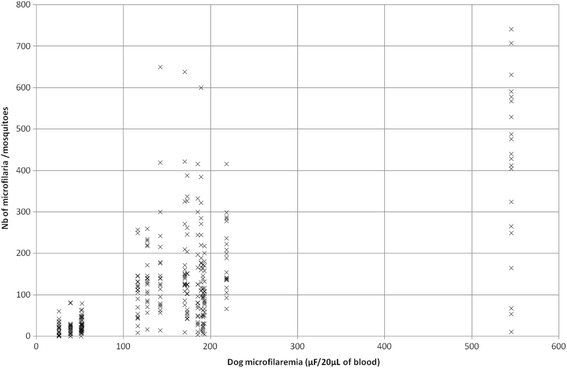
Relationship between canine microfilaremia and uptake of microfilariae by blood-fed mosquitoes from all dogs before treatment (*n* = 6) and in the control group at weekly intervals over 1 month (*n* = 3)

In the control group and before treatment for all individuals, the uptake of microfilariae by mosquitoes during blood-feeding was variable but successful (Fig. 1 and Table 5). There were from 0 to 742 microfilaria per mosquito, the average microfilarial load was 119.7 in the control group and 95% of the engorged mosquitoes had microfilariae (data not shown). In the treated group, since no mosquitoes fed on dogs on Day 7, none was dissected. The few (*n* = 22) mosquitoes that fed on the treated dogs between Days 14 and 28 were not dissected in order to assess their survival and potential for transmission of heartworm.

**Table 5:** *Dirofilaria immitis* microfilaria uptake by *Aedes aegypti* mosquitoes blood-fed on infected dogs untreated or topically treated with DPP on Day 0

Control			DPP^a^	
Day	Fed Mosquitoes Dissected/Total^b^	Microfilariae/ Mosquito^c^	Fed Mosquitoes Dissected/ Total	Microfilariae/ Mosquito
−7	60/208	57.5 ± 83.4 (0-416)	60/180	153.1 ± 218.8 (0-742)
7	60/206	98.6 ± 81.0 (7-316)	0/0	0
14	60/224	155.5 ± 109.8 (15-650)	Na^d^/6	–
21	60/177	96.0 ± 59.4 (5-258)	Na/8	–
28	60/203	191.1 ± 120.4 (10-638)	Na/8	–

^a^DPP: dinotefuran + permethrin + pyriproxyfen (Vectra® 3D)

^b^Fed mosquitoes sampled from three exposure cages (20 per dog) during exposure for dissection and microfilarial count /total fed mosquitoes count

^c^Average number ± standard deviation (min-max) of microfilariae per mosquito dissected

^d^Fed mosquitoes were not dissected and were incubated for survival assessment

### Knock-down efficacy

In the control group before and after Day 0 and in the treated group before treatment, the geometric mean number of live mosquitoes after the 1 h exposure ranged from 66.8 to 81.8, and there were no moribund mosquitoes (Table 4). For 1 month after treatment, the geometric mean number of live mosquitoes in the treated group ranged from 0 on Day 7 to 3.3 on Day 21. The moribund and live mosquitoes were incubated for survival assessment. The knock-down efficacy of DPP 1 h after each infestation ranged from 95.5% to 100% over 1 month (Table 4). It is also noteworthy that compared with the control group, mosquitoes in the treated group spent a relatively small amount of time in contact with the treated dogs.

### Insecticidal efficacy and survival of mosquitoes

In the control group, throughout the study and before treatment in all individuals in both groups, the survival rate of the mosquitoes during the incubation decreased from 74.7% to 94.8% on Day 1 to 6.9% to 26.6% on Day 16 (Table 6). In the treated group, the survival rate of the mosquitoes during the incubation decreased from 0 to 4.0% on Day 1 to 0 to 0.4% on Day 16. Of the cumulative total of 1341 mosquitoes that were exposed to the treated dogs during the four exposure periods, a total of 22 mosquitoes fed on these dogs; and all were dead within 72 h (data not shown). The survival of the fed mosquitoes appeared to be related to the microfilaremia level of the donor dogs, with a higher death rate in mosquitoes that fed on dogs with the highest microfilarial counts; but these data were not analyzed statistically.

**Table 6:** Average survival rate of *Aedes aegypti* female mosquitoes (%) assessed daily during a 16-day incubation period after 1 h exposure to control or DPP-treated microfilaremic dogs infected with *Dirofilaria immitis*

Infestation		Duration of Incubation (days)															
Group	Exposure Day	1	2	3	4	5	6	7	8	9	10	11	12	13	14	15	16
Control	−7	92.7	76.7	65.7	55.0	50.3	49.2	46.4	42.3	40.4	38.7	37.1	33.4	30.6	27.7	24.0	22.9
	7	94.8	83.0	57.6	47.2	43.7	41.5	39.8	38.0	36.2	36.2	33.6	27.9	23.8	20.6	19.3	16.2
	14	82.5	48.8	36.1	28.6	26.6	25.8	25.0	24.6	23.7	20.4	19.6	16.8	15.6	14.7	13.9	13.5
	21	94.5	77.8	56.9	52.8	48.7	47.8	44.2	42.8	37.9	36.5	36.1	33.4	31.1	28.9	27.0	26.6
	28	74.7	43.8	26.5	21.9	21.4	19.5	16.7	14.8	12.5	12.0	10.6	10.2	8.3	7.3	6.9	6.9
DPP^a^	−7	91.0	79.2	71.6	62.9	56.3	53.8	49.2	46.2	43.2	38.0	36.6	34.1	27.4	25.1	20.6	19.6
	7	0.0	0.0	0.0	0.0	0.0	0.0	0.0	0.0	0.0	0.0	0.0	0.0	0.0	0.0	0.0	0.0
	14	0.8	0.8	0.8	0.8	0.8	0.8	0.8	0.8	0.8	0.8	0.8	0.8	0.8	0.4	0.4	0.4
	21	4.0	1.8	1.4	1.4	1.4	1.4	1.4	1.4	1.4	1.4	1.4	0.7	0.7	0.7	0.4	0.4
	28	1.1	0.4	0.0	0.0	0.0	0.0	0.0	0.0	0.0	0.0	0.0	0.0	0.0	0.0	0.0	0.0

^a^DPP: dinotefuran + permethrin + pyriproxyfen (Vectra® 3D)

### L3 development blocking efficacy

In the control group before and after Day 0 and in the treated group before treatment, a total of 222 mosquitoes survived the 16-day incubation period. A total of 132 fed mosquitoes were dissected (Table 7), and 121 (91.6%) of them had at least one L3. In the control group, there was an average of 12.3 L3 per engorged mosquito; and up to 39 L3 were found in a single fed mosquito (data not shown). After treatment with DPP, only two unfed mosquitoes survived the incubation period; and no L3 were found in them when they were dissected.

**Table 7:** Average larval (L3) load of blood-fed and nonblood-fed *Aedes aegypti* female mosquitoes exposed for 1 h to microfilaremic dogs infected with *Dirofilaria immitis* incubated for 16 days prior to dissection

Control						DPP^a^			
Exposure Day	Dissection Day	Fed Mosquitoes^b^	Unfed Mosquitoes	L3/Fed Mosquito	L3/Unfed Mosquito	Fed Mosquitoes	Unfed Mosquitoes	L3/Fed Mosquito	L3/Unfed Mosquito
−7	9	24	22	8.3	0.8	22	12	8.2	0.0
7	23	29	13	11.1	0.5	–^c^	–	–	–
14	30	19	15	14.6	3.1	0	1	0	0.0
21	37	22	26	12.0	0.3	0	1	0	0.0
28	44	16	Na^d^	18.3	0	0	0	Na	Na

^a^DPP: dinotefuran + permethrin + pyriproxyfen (Vectra® 3D)

^b^Cumulated number of surviving mosquitoes (fed or unfed) dissected after the 16-day incubation period

^c^No blood-fed or surviving mosquitoes on treated dogs after exposure on Day 7

^d^No surviving mosquitoes after the 16-day incubation period

## Discussion

### Methodological considerations

The dog/mosquito exposure model used in this study was considered successful since the mosquitoes that were released with the untreated dogs were able to feed, take up microfilariae and allow L3 to develop during a 16-day incubation period. A total of only 222 mosquitoes survived the 16-day incubation period, and 132 had engorged on untreated dogs. A total of 121 (91.6%) of these mosquitoes had at least one and as many as 39 L3. In a natural environment, these vector mosquitoes could spread heartworm from canine reservoirs to other susceptible hosts.

Interestingly, we observed that the mosquitoes that fed on the most highly microfilaremic dogs died more quickly than those that fed on the dogs with more moderate microfilaremia. Such differences could be explained by a limit in the parasite load that can be tolerated by the mosquito vectors. This is consistent with previous observations performed on mosquitoes feeding on *D. immitis–*microfilaremic dogs [2, 11] as well as mosquitoes feeding on *Wuchereria bancrofti–*infected, microfilaremic human hosts [12]. It was indeed demonstrated that the parasite load in mosquitoes was a risk factor of vector survival [12], and “hidden carriers” with low microfilaremia were suspected of playing a major reservoir role [13]. In a natural environment, the parasite load of mosquitoes with *D. immitis* usually ranges between one and eight L3 per mosquito [14]. In the present experiment, up to 39 L3 were able to develop in a single mosquito and survive the 16 days of incubation. A high parasite load of mosquitoes has already been reported in experimental conditions: 62 *D. immitis* L3 were found in a single female *Aedes notoscriptus* after 10 days of incubation [11].

### Efficacy against mosquitoes (*A. aegypti*)

The anti-feeding efficacy of DPP in this study ranged from 95.8% to 100% and was consistent with previous measurements on the same mosquito species, with efficacy ranging from 87.0% to 94.0% [8]. This high-level repellency (anti-feeding) effect was also reported against other flying insects, such as *Culex pipiens* mosquitoes [15], *Phlebotomus perniciosus* sand flies [16] and *Stomoxys calcitrans* biting flies [17]. The knock-down effect of 98.0% to 100% observed 1 h after exposure is also in line with previous data obtained with DPP showing 93.0% to 100% knock-down effect [8].

Although mosquitoes in both groups were allowed access to the dogs for up to 1 h, it was noted that those in the treated group spent a relatively short period of time in direct contact with the treated dogs, when compared with controls. The few mosquitoes that were able to feed on the treated dogs died within 72 h of incubation. While permethrin is the active ingredient providing repellency (anti-feeding), dinotefuran is known as a fast-acting insecticide and was identified as a promising active agent against resistant mosquitoes [6]. When dinotefuran and permethrin are combined at the ratio found in DPP, synergy occurs; and the permethrin improves the action of dinotefuran at the synaptic level in insects [18]. In DPP, this synergy provides an increased efficacy against insect parasites. The lethal anti-feeding effect of DPP was expected to block the transmission of *D. immitis* from infected microfilaremic dogs to the mosquito vectors.

### Efficacy against heartworm (*D. immitis*)

Only a few mosquitoes (*n* = 22) were able to feed on the treated dogs during the first month after treatment. Since priority was given to assessment of the anti-transmission efficacy, these insects were not killed for dissection but rather were incubated for assessment of their survival and potential development of L3. None of the mosquitoes that fed on treated dogs survived for more than 72 h of incubation. A few (n = 2) nonblood-fed mosquitoes exposed to treated dogs remained alive during the 16-day incubation and were dissected. None of them exhibited L3. It is reasonable to assume that the high mosquito repellency and insecticidal efficacy levels obtained in our study are independent of the resistance status of the filarial isolate, as similar values were reported in a study with *A. aegypti* mosquitoes and DPP where there was no heartworm component [8]. However, studies with other heartworm strains (ie, macrocyclic lactone-resistant or -susceptible) are yet to be done. Transmission-blocking strategies target the parasite within the insect vector and are expected to reduce the prevalence of infection in endemic communities. Such consequences have already been proven to be successful experimentally against malaria (*Plasmodium berghei*) and its anopheline vector (*Anopheles stephensi*) [19]. Our results with another dog/mosquito exposure model indicate that DPP would represent a reliable weapon for transmission-blocking of *D. immitis* L3 to dogs [20]. The treatment of reservoir dogs is indeed expected to reduce the risk of infection in the neighborhood and anywhere the dog travels.

## Conclusions

The xenodiagnosis-type dog/mosquito laboratory model used successfully in this study has also been shown to be useful in assessing the transmission of *D. immitis* L3 from infected mosquitoes to dogs and other hosts. The topical formulation of DPP used in this study was more than 95% effective in repelling and killing mosquitoes for 28 days after treatment. Thus, it was more than 95% effective in blocking the acquisition of microfilariae by mosquitoes; and, because all of the few mosquitoes that fed on the treated, microfilaremic dogs died within 3 days, it was 100% effective in blocking subsequent transmission of any L3 to dogs or other susceptible hosts. Repellent and insecticidal properties of ectoparasiticides could contribute substantially to reducing the risk of heartworm transmission, even those heartworm biotypes resistant to macrocyclic lactone preventives or any other type of preventive. A multimodal approach to the prevention of heartworm, which reduces populations of mosquitoes, prevents mosquito biting, kills mosquitoes and includes the monthly or biannual administration of a macrocyclic lactone preventive, should be strongly encouraged.

## Abbreviations

DPP: dinotefuran-permethrin-pyriproxyfen
MF: microfilariae
